# Structural and procedural barriers to health assessment for asylum seekers and other migrants – an explorative survey in Sweden

**DOI:** 10.1186/s12913-018-3588-6

**Published:** 2018-10-23

**Authors:** Robert Jonzon, Pille Lindkvist, Anna-Karin Hurtig

**Affiliations:** 1 The Public Health Agency of Sweden, Nobels väg 18, SE-171 82 Solna, Sweden; 2 Deparment of Public Health and Clinical Medicine, Umeå University, Umeå, Sweden; 3 Center for Family Medicine (CeFAM), Karolinska Institute, Stockholm, Sweden

**Keywords:** Health system, Health assessment, Asylum seekers, Migrants, Sweden

## Abstract

**Background:**

Health assessments (HAs) for newly arrived asylum seekers have become a regular practice in most EU countries, but what is performed, how they are organized, and whether it is mandatory or not to attend varies between countries. Swedish national statistics have shown that only about 45% of asylum seekers attend the optional HA offered upon their arrival in Sweden. There are significant variations among Sweden’s 21 counties, ranging from 20 to 90%. The reasons for the low attendance have not yet been fully explored, though there are indications of structural weaknesses within the healthcare system. This study aimed to identify variations in policies and implementation of HAs targeting asylum seekers and other migrants. The study analyzes the structure and processes in different Swedish counties and discusses how this might influence the coverage.

**Methods:**

This research project had an exploratory quantitative descriptive design applying a cross-sectional survey based on two structured questionnaires. Descriptive statistics were performed to summarize the data.

**Results:**

The number of healthcare centers in each county that carried out HAs on asylum seekers varied independently of the size of the county. Variations in regard to structure, organization, processes, and performance monitoring of the HA process also appeared diverse, and these were in some cases also reported differently by administrators and healthcare professionals in the same county. Most commonly, the HAs were carried out in ordinary health centers, though some counties presented alternative solutions on how to organize the HAs.

**Conclusions:**

There seems to be no coherent national system for carrying out HAs on asylum seekers in Sweden. The structure, organization, processes, and outcomes vary between the counties, and the reasons for the low coverage of HAs appear to be multifaceted.

## Background

Immigration to Sweden has since the 1970s predominantly consisted of asylum seekers and their families [1, 2]. Even before the current vast influx of migrants, mainly due to the armed conflict in Syria, Sweden has been a major recipient country within the European Union (EU) [3]. For many years, approximately 30,000 individuals annually have applied for asylum in Sweden, but in 2015 more than 162,000 asylum seekers arrived [4]. Human rights principles guide the asylum process, and asylum seekers’ right to health has been particularly emphasized among those principles [5–7]. Health assessments (HAs) for newly arrived asylum seekers have become a regular practice in most EU countries, but these vary in terms of content, whether they are voluntarily, and how they are organized [8].

In Sweden, healthcare for asylum seekers has been limited to a voluntary HA and care that cannot be postponed. On 1 July 2008, this practice was encoded into law [9]. Entitlements to the HA are primarily made on the migrant’s legal status, not on their health needs or on epidemiological data. However, the national strategy against HIV emphasizes the importance of the HA for newly arrived migrants, soon after arrival, in order to detect possible HIV and other communicable diseases at an early stage [10]. Even if data from recent studies [11, 12] show that asylum seekers consider the HA to be an important offer to them, only about 45% attend. This is based on national data and calculated on a national level annually [13]. On the county level, significant variations are seen in the number HAs carried out, ranging from approximately 20% to 90% of new immigrants. The reasons behind the low rate are not yet clear, although explanations related to structural and organizational shortcomings have been suggested [14].

In Sweden, each county council, and municipalities that are not part of a county, are responsible for providing healthcare of good quality and equal access to the people residing within the county [15]. For the most part, this also applies to migrant populations [9]. However, the principle of local self-governance gives the county councils and regions sovereignty to structure and organize their own healthcare resources in accordance to local conditions. This in turn might lead to variations across the country. The Swedish national healthcare services are largely tax financed and can be carried out either within the public or private sector [15–17].

The Swedish National Board of Health and Welfare has in a report [18] indicated that the HA should aim at identifying health problems in asylum seekers and other migrants and should be used to carry out effective measures of infectious disease prevention and control. It also aims to give the newcomers information about the Swedish healthcare system. The same report [18] also stated that almost all county councils lacked effective procedures to reach all asylum seekers. There was also a lack of routines to ensure good quality and follow up of the services that are carried out. Hence, it seems as if Sweden so far has not found an optimal structure for the HAs targeting asylum seekers and other migrants. Further research is needed to identify how to organize the health care targeting migrants in the best way, and particularly the HA services.

The Donabedian model suggests that the quality of health service can be assessed from three aspects – structure, process, and outcome [19]. The structural aspects of health service consist of physical characteristics (e.g. resources and management) and staff characteristics (e.g. skill and teamwork). Process is defined as the way of providing clinical and interpersonal care to the patient. Outcome is the end product of interactions between structure and process, which can be measured by health status indicators and user evaluations.

This study aimed to identify variations in policies and implementation of HAs for asylum seekers in Sweden. The study analyzes the structures and processes of HAs in different Swedish counties and discusses how this in turn might influence the coverage.

## Methods

### Design

This study had an exploratory quantitative descriptive design using a cross-sectional survey, and it was based on two structured questionnaires that were pre-tested [20]. The pretest was carried out face to face and individually with four nurses who had significant experience in both administration in relation to HAs for migrants as well as performing the HA as such. The overall questionnaires with their preliminary themes were discussed along with each question separately. At the fourth piloting, nothing new came up and it was concluded that the questionnaire needed no further changes.

### Study setting and participants

The basis of the Swedish healthcare system is generally referred to as primary healthcare, consisting of local health centers. At this level, health promotion and preventive actions play an important role in addition to the care and treatment of illnesses that do not require specialized medical care, which is provided by hospitals. The health centers are usually staffed by general practitioners (GPs), nurse practitioners, physiotherapists, and occupational therapists. It is at these local health centers that the asylum seekers’ HAs take place. As for all Swedish healthcare, there are different sets of rules and regulations that apply to healthcare targeting migrants [9, 15, 21].

The participants of this study were the appointed officers in each of the 21 county councils who were in charge of administrative matters in relation to healthcare for asylum seekers. Clinicians, including both doctors and nurses, performing HAs of asylum seekers at each of the health centers appointed to carry out HAs, were also involved.

### Data collection

Two different questionnaires were distributed, one targeting administrative staff and the other aimed at clinicians. The questionnaires had considerable content overlap, thus allowing for comparison of the results. The questions covered the demographic characteristics of the participants and asked their opinions on healthcare organization and structure, competencies and responsibilities, performance management, information and communication, and performance monitoring.

Questionnaire A was addressed to all appointed administrators (*n* = 21) responsible for matters concerning healthcare for asylum seekers in the 21 county councils. Because one main focus of the study was to describe variations among the county councils in terms of organization, structure, and the way HAs are carried out, all 21 county councils were included.

Questionnaire B was addressed to those primary healthcare centers within the 21 county councils that were appointed to carry out HAs on migrants, according to information given by each of the county councils themselves. All units (*n* = 785) were included in this survey. The questionnaire was to be answered by any healthcare personnel involved in the HA of asylum seekers.

The postal questionnaires were sent out in April 2010, and the closing date was set to August 31 the same year. In the meantime, two reminders for both of the questionnaires were sent out.

Both questionnaires mainly contained questions with fixed response alternatives, although some questions also had open-ended response alternatives.

Questionnaire A. The questionnaire, together with instructions, was sent to the appointed administrators, and 20 (95.2%) out of the total 21 counties responded and returned the questionnaire.

Questionnaire B. The questionnaire, together with instructions, was sent to the head of each of the 785 primary healthcare centers. Only one questionnaire per healthcare center was to be filled in and sent back. Each questionnaire was coded and returned anonymously. Of the 785 questionnaires distributed, 315 (40%) were answered and returned. These 315 were all included in the analysis.

### Analysis of missing questionnaires

Questionnaire A. One of the county council’s central administration was missing. In addition to two written reminders, a telephone call was made to confirm that the questionnaire was received*.*

**Fig. 1:**
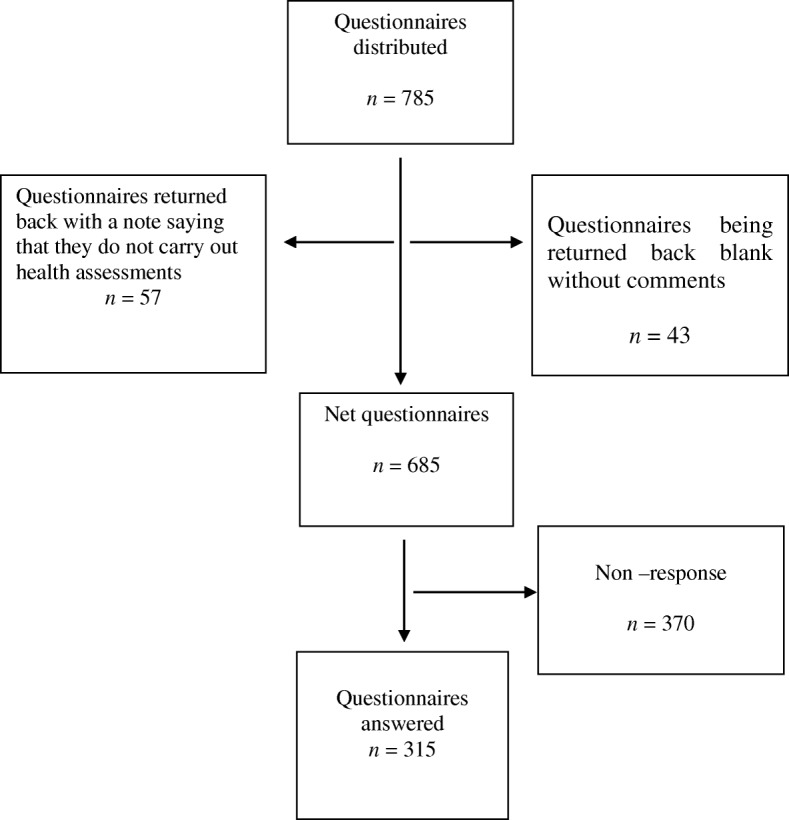
Flow diagram of respondents of the survey and eligibility for the study

Questionnaire B. Among the 470 non-responders, 57 returned the questionnaire blank with a note saying that they did not work within the concerned field, which led us to suspect that there was an over coverage. Another 43 returned the questionnaire blank, without a note, and the remaining 370 did not reply at all (Fig. 1).

In order to be able to explain the relatively high number of non-responding healthcare centers, a county-stratified random sample was drawn. The sample size calculations assumed a 35% chance that a non-responding healthcare center was not providing HAs on asylum seekers. Furthermore, a precision of 5% for the confidence interval for the estimate of the total number of non-providing healthcare centers was assumed. The calculations yielded a sample size of 213 of the 470 health centers that did not participate. The 213 were contacted by telephone and almost all (199) said the reason for not responding was that they, at the time of the survey, did not carry out HAs on migrants. Based on this figure, we estimated the proportion of the over coverage to be 94% [95% CI (91% to 96%)], which we consider to be a significantly high over coverage, and thus these are not true non-responders. The explanation for this is that the inclusion criterion was health centers appointed to carry out HAs on asylum seekers, but in reality only part of them actually carried out HAs. Thus, only those health centers that in fact carried out HAs should have been selected to participate, instead of all that were appointed to carry out these services.

### Analysis strategy

Descriptive statistics were performed to summarize and analyze the data (20). Frequency distributions were calculated using the statistical package Stata 13.0. The analysis was inspired by the Donabedian framework [19]. The answers to the open-ended questions were analyzed by means of qualitative content analysis [22].

## Results

### Characteristics of the participants

Sixteen of the administrators (80.0%) were women, and 247 of the healthcare professionals (78.4%) were women. Among the healthcare professional respondents, 217 (69.0%) were general nurses and community health nurses, and 70 (22.2%) were GPs.

Sixteen of the administrators (80.0%) did not have special education or training in relation to administering healthcare targeting migrants, while 4 (20.0%) indicated that they had. Among the doctors and nurses, 53 (16.8%) indicated having had post-graduate training or specialization in healthcare in relation to migration, refugees, international health, or similar, while 255 (81.0%) had no such training.

Though not a formal training, 55 (17.5%) of the healthcare personnel had experience from professional work abroad within the healthcare sector. Among the administrators, none indicated such experience.

Sixteen administrators (80.0%) were born in Sweden, and 3 (15%) were born in another country. A similar distribution was seen among the healthcare personnel, where 286 (90.8%) were born in Sweden and 25 (7.9%) were born abroad.

Among the administrators, 10 (50.0%) had been working 5 years or less in administration related to migrants’ healthcare, 6 (30.0%) of them between 6 and 10 years, and 3 (15.0%) for more than 10 years. Among the healthcare personnel, 161 (51.1%) had been carrying out HAs on asylum seekers for 5 years or less, 51 (16.2%) between 6 and 10 years, and 45 (14.3%) for more than 10 years. However, as many as 58 (18.4%) respondents did not respond on this issue (Table 1).

**Table 1:** Characteristics of the participants

	Administrator at regional/county level *n* = 20 (%)	Health care personnel at health centers *n* = 315 (%)
Sex		
Female	16 (80.0)	247(78.4)
Male	4 (20.0)	58 (18.4)
Missing	0	10 (3.2)
Work position		
Administrator	14 (70.0)	
Economist	3 (15.0)	
Manager	1 (5.0)	
Investigator	1 (5.0)	
Physician		70 (22.2)
Nurse/community nurse		217 (69.0)
Head of unit / Manager		42 (13.3)
Missing	1 (5.0)	26 (8.3)
Country of birth		
Sweden	16 (80.0)	286 (90.8)
Europe (except Sweden)	3 (15.0)	16 (5.1)
Outside Europe		9 (2.9)
Missing	1 (5.0)	4 (1.3)
Special training in relation to migrants’ health		
Yes	4 (20.0)	53 (16.8)
No	16 (80.0)	255 (81.0)
Missing		7 (2.2)
Experience of similar work abroad		
Yes		55 (17.5)
In a European country		27 (49.1)
In a country outside Europe		26 (47.3)
Missing		2 (3.6)
No	17 (85.0)	255 (81.0)
Missing	3 (15.0)	5 (1.6)
Number of years in the present or similar position		
1–5 years	10 (50.0)	161 (51.1)
6–10 years	6 (30.0)	51 (16.2)
More than 10 years	3 (15.0)	45 (14.3)
Missing	1 (5.0)	58 (18.4)

### Organization and structure

The number of healthcare centers in each county that carried out HAs on asylum seekers varied between 1 unit and more than 10 units, regardless of the size of the county.

Six (30.0%) of the 20 administrators reported that in their county the health centers carrying out HAs were run by the county council, 1 (5.0%) by private entrepreneurs, and 11 (55.0%) by both county councils and private entrepreneurs.

The healthcare personnel specified within what setting and structure they carried out HAs. The results reveal that most HAs took place at ordinary healthcare centers (Table 2).

**Table 2:** The setting for the HA

The setting and structure where the health assessments were carried out	*n* = 315
An ordinary healthcare center	282
A separate and special unit within the frame of an ordinary healthcare center	19
A health center exclusively targeting asylum seekers (and other migrants)	2
A mobile unit	1
Missing	11

Nineteen administrators (95.0%) indicated that in their county there was a centralized support function at the county level in relation to HAs for migrants. The competences represented in this function were administrative, 17 (85.0%); financial, 18 (90.0%); legal, 5 (25.0%); and medical/nursing, 8 (40.0%).

Ninety-eight (31.1%) of the healthcare professionals indicated that their county had a centralized support function for those working in the healthcare centers carrying out HAs on asylum seekers. However, 50 (15.9%) denied having such a resource, and as many as 126 (40.0%) did not know whether the county in which they were working had such a support function or not.

The respondents, both administrators and healthcare personnel, have in their answers to the open-ended questions given examples on their county’s particular way of organizing the HA services:

“In our county there is only one specialized unit for health assessments.”

“One centralized healthcare center constitutes the base, from where mobile teams reach out to all parts of the county where asylum seekers reside and where the health assessments are performed.”

“That the assignment to carry out health assessments is geographically spread across the county to several health centers.”

### Competencies, responsibilities, and team support

In the narratives of the open-ended questions, the administrators described their position and responsibilities. They commonly reported being located at the central county council office. Some indicated that they themselves constituted the central support function, while for others this was a shared duty among several individuals, either full time or part time. Their main areas of concern were administration and economics, but also legal and policy issues. They comprised a link between the migration authority and the healthcare centers, which included obtaining personal data from the migration authority on newly arrived asylum seekers. Similarly, they were responsible for collecting data on the HAs being carried out and reporting this information to The Swedish Association of Local Authorities and Regions (SALAR) for the annual national statistics. However, these administrative duties and responsibilities were in some cases, and to varying extents, assigned to the staff at the health care centers (Table 3).

**Table 3:** Administrative duties

	Administrator at county level *n* = 20 (%)	Health care personnel at local health centers with administrative duties *n* = 192 (%)
Type of administrative duties		
Obtain data from the migration authority on recently arrived asylum seekers	16 (80.0)	109 (56.8)
Notify healthcare centers on recently arrived asylum seekers	11 (55.0)	35 (18.2)
Invite recently arrived asylum seekers to the health assessment	1 (5.0)	145 (75.5)
Report to the migration authority in order to obtain state refunds for health assessments carried out	13 (65.0)	47 (24.4)
Report to SALAR on health assessments carried out	17 (85.0)	11 (5.7)

Besides the core responsibility, namely carrying out HAs, as all 315 (100.0%) physicians and nurses did, 159 (50.5%) indicated that they also provided general healthcare to asylum seekers and 192 (61.0%) that they also had administrative duties besides healthcare. These responsibilities were mainly confined to communication with the migration authority on obtaining personal data of recently arrived asylum seekers, data that were a prerequisite for being able to invite them to the HAs, and 109 (56.8%) had this responsibility and 145 (75.5%) were responsible for inviting asylum seekers to the HA. Further, of those having administrative responsibilities, 45 (23.4%) indicated that they were responsible for reporting back to the migration authority on the number of HAs that were carried out in order to receive a financial refund from the state. Similarly, 11 (5.7%) were responsible for giving an account to SALAR, who produces annual national statistics on the number asylum seekers being assessed.

The physicians and nurses had limited access to professional supervision at work. A total of 63 (20.0%) were offered such a resource, but the majority, 205 (65.1%), were not.

Sixty-six (21.0%) of the respondents among the healthcare professionals indicated that they had access to professional networks in relation to migrant health, while 99 (31.4%) did not have such a network, and 121 (38.4%) were not able to state whether such a network existed or not. Among the administrative respondents, 8 (40.0%) indicated having professional networks and cooperation across county borders, 10 (50.0%) did not, and 2 (10.0%) did not know whether such a network existed or not. Examples of cooperation given by the respondents included networking meetings on a regular basis arranged by SALAR and meetings for sharing experiences between the administrators working in the three major cities; Stockholm, Gothenburg, and Malmö.

### The characteristics of clinical staff carrying out the health assessment

The healthcare personnel reported that for the most part the initial health interview was carried out by nurses, 222 (70.5%), while 57 (18.1%) indicated it was carried out by a GP. The assessment that followed from the health interview was reported to be carried out on more equal terms, and 151 (47.9%) reported that this was carried out by nurses and 138 (43.8%) reported that this was carried out by GPs.

Among the healthcare personnel, 211 (67.0%) perceived having the qualifications needed to do the work in a satisfactory way. However, 44 (14.0%) felt that they did not have the necessary competence.

The healthcare personnel (the GPs and nurses) carrying out HAs on asylum seekers had to some extent different access to professional consultants without a formal referral procedure. The respondents reported to what extent they had access to different kinds of specialists by telephone, on site, or both. Further they indicated whether they considered such support to be sufficient or not (Table 4). The consultants, independently of what kind of specialist, were most commonly available by telephone and rarely on site. Few respondents considered the access to consultants to be sufficient. The access to psychiatrists and psychiatric nurses was rated the most insufficient.

**Table 4:** Access to consultants

	Available by telephone	Available on site	Available by telephone and on site	Considered sufficient
Kind of consultants	Health care personnel *n* = 315 (%)			
Pediatrician	95 (30.2)	22 (7.0)	20 (6.4)	81 (25.5)
Gynecologist / obstetrician	86 (27.3)	8 (2.5)	3 (0.1)	59 (18.3)
Psychiatrist	86 (27.3)	8 (2.5)	7 (2.2)	40 (12.3)
Psychiatric nurse	76 (24.1)	17 (5.4)	21 (6.7)	56 (17.5)
Dentist	72 (22.9)	23 (7.3)	25 (7.9)	84 (26.4)

### The health assessment: Process and content

The starting point for the HA process is the invitation, which the healthcare providers by law are obliged to communicate to the asylum seekers. In this study, 192 (61.0%) of the healthcare personnel reported using a written invitation in Swedish, 13 (4.1%) used a written invitation in English, and 26 (8.3%) used a written invitation in the migrants’ native language, and 101 (34.6%) reported that the invitation contained explicit information about the HA being optional, while 129 (40.1%) reported that this was not mentioned in the invitation. A total of 256 (81.3%) of the healthcare personnel reported that an interpreter was involved during the actual HA, and of these 144 (45.7%) indicated that the interpretation was done by means of telecommunication and 114 (36.2%) reported that they used the interpreter on site at the healthcare center.

Some of the respondents commented:

“With constant delays in our processes, and the EBO* policy, the written invitation does not reach the asylum seeker and is returned to us”.

*EBO (Swedish: Eget BOende) is an option for asylum seekers to decline the accommodation provided by the Migration Agency, and instead on one’s own find a suitable accommodation. Many choose to live with friends or relatives, at their own expense, while they wait for a response to their application for asylum. Some may move from one address to another, and sometimes the valid address at any given time is not communicated to the Migration Agency.

“The county has a vast geographical area. Some migrants live far away from the healthcare center and have limited access to public transportation services”.

The content of the HA varied among the counties, as reported by the respondents. Among the topics presented below (Table 5), several are regulated in national guidelines and must be covered within the HA. However, only 201 (63.8%) indicated that they, in connection to the HA, gave information about the Swedish healthcare system, leaving many newcomers without this essential knowledge. Even if not completely, issues related to contagious diseases seemed to be prioritized, and 236 (74.9%) of the informants indicated that vaccination status was always checked in children. Tests for TB and HIV were considered a rule by 234 (74.3%) and 248 (78.8%) of the informants, respectively. Although information booklets on HIV and other STIs have been produced and translated into many languages by the Swedish Public Health Agency, only 91 (28.9%) reported having these available at their health center. Notably, as presented in Table 5, there is a significant proportion missing values on each of the topics covered on the contents of the HA.

**Table 5:** The contents of the health assessment

	Agree totally	Agree partly	Disagree partly	Disagree totally	Missing
Issues in relation to the health assessment	*n* = 315 (%)				
The health assessment is primarily guided by the individual asylum seeker’s health needs, rather than by checklists	37 (11.8)	126 (40.0)	51 (16.2)	36 (11.4)	65 (20.6)
Information about the Swedish health care system is routinely given in connection with the health assessment	110 (34.9)	91 (28.9)	34 (10.8)	19 (6.0)	61 (19.4)
Vaccination status is always checked with children	219 (69.5)	17 (5.4)	7 (2.2)	3 (1.0)	69 (21.9)
Vaccination against Rubella is always offered to fertile women	37 (11.8)	48 (15.2)	31 (9.8)	101 (32.1)	98 (31.1)
Sexual health issues are routinely checked within the health assessment	45 (14.3)	100 (31.8)	60 (19.1)	41 (13.0)	69 (21.9)
TB test is a rule at the health assessment	192 (61.0)	42 (13.3)	12 (3.8)	12 (3.8)	57 (18.1)
HIV test is a rule at the health assessment	228 (72.4)	20 (6.4)	4 (1.3)	6 (1.9)	57 (18.1)
Information on how to prevent HIV and other STIs is routinely communicated	37 (11.8)	76 (24.1)	72 (22.9)	65 (20.6)	65 (20.6)
Information booklets on HIV and other STIs is available at the healthcare center	39 (12.4)	52 (16.5)	51 (16.2)	107 (34.0)	66 (21.0)

### Managing and monitoring quality

In order to harmonize services and create an equitable healthcare system, the need for steering documents and guidelines is commonly recognized. In this study, only 14 (70.0%) administrators and 158 (50.2%) healthcare personnel reported that such steering documents, at the county level and specifically written to guide healthcare targeting asylum seekers and other migrants, were in place.

It is also widely recognized that in order to secure high-quality healthcare, continuous and systematic quality assurance work needs to be undertaken. Among the administrators, 6 (30.0%) reported having such work implemented in their county. Similarly, methods and measures used in healthcare need to be continuously evaluated, developed, and improved, which also applies to the HA services targeting asylum seekers, and 9 (45.0%) administrators reported that such systematic method improvement work is implemented in their county. From those who indicated having such ongoing work in their county, examples were given in the responses to the open-ended questions. Almost exclusively, the examples were about improving guidelines and checklists related to the HA. Among the healthcare professionals, 217 (68.8%) indicated having written guidelines on the content and performance of the HA.

The HA should ideally take place as soon as possible after an individual arrives in Sweden. This requires reduction in the lead times in the process leading up to the HA, and the respondents estimated three crucial lead times (Table 6). Noteworthy is that more than one third of the respondents were either not able to estimate these lead times or did not respond to the question.

**Table 6:** Estimated lead times

	Administrator at county level *n* = 20 (%)		Health care personnel at local health centers *n* = 315 (%)
	Estimated average time from the registration of an asylum application at the migration authority until the county council is notified of the application	Estimated average time from when the county council is notified about a newcomer until the healthcare center is notified	Estimated average time from when the health care center is notified until the health assessment takes place
< 1 month	1 (5)	4 (20)	90 (28.6)
1 to < 2 months	4 (20)	2 (10)	71 (22.5)
≥2 months	5 (26)	6 (30)	38 (12.1)
Do not know	8 (40)	6 (30)	57 (18.1)
Missing	2 (10)	2 (10)	59 (18.7)

Procedures to follow up the HA process need to be in place, and the respondents were asked if such prerequisites were established that allowed for annual follow ups of asylum seekers who had an HA. Among the administrators, 10 (50.0%) indicated that they had what was needed to do such annual follow ups, and 135 (42.9%) healthcare personnel stated having such procedures in place.

The respondents were also asked to indicate the proportion of asylum seekers having arrived during the previous year, in relation to all asylum seekers in the county and in the area assigned to the local healthcare center, respectively, who had an HA carried out during the previous year in the county as a whole (administrators at county level) and in the healthcare center were they worked (healthcare personnel) (Table 7). There is a significantly high number missing values on this issue among both the administrators and the healthcare personnel, 60.0% and 72.4% respectively. If to the missing values and the “Do not know”-answers are added it shows that less than 20% of the healthcare personnel were able to estimate the proportion of asylum seekers having had an HA carried out during the previous year in the healthcare center where they were working.

**Table 7:** Estimated proportion asylum seekers having undergone the HA

	Administrator at county level *n* = 20 (%)	Healthcare personnel at local health centers *n* = 315 (%)
	Proportion of asylum seekers having had a health assessment carried out during the previous year in the county as a whole	Proportion of asylum seekers having had a health assessment carried out during the previous year in the local healthcare center
< 25%	2 (10.0)	7 (2.2)
25–49%	3 (15.0)	0
50–74%	2 (10.0)	8 (2.5)
75–100%	1 (5.0)	46 (14.6)
Do not know	0	26 (8.6)
Missing	12 (60.0)	228 (72.4)

Finally, the respondents (healthcare personnel) were asked whether a specific follow up was done in order to find out the reasons why asylum seekers did not turn up for an HA despite an invitation or declared their intent not to attend. Among those who replied, 31 (9.8%) answered that they did try to determine the reason behind the dropouts, 176 (55.9%) did not, and 48 (15.2%) did not know if such inquiries were made.

## Discussion

The exploratory research presented here aimed to identify variations in policies and practice, as well as organizational differences between Swedish counties, relating to asylum seekers’ HAs and how these potential differences might influence the number of asylum seekers being assessed. The study was felt to be urgent and highly relevant in light of the fact that, for a number of years, fewer than 50% of all asylum seekers in Sweden have undergone the HA [13, 23, 24]. In addition, no effort has been made to find out the reason behind this. In an attempt to improve and maybe even solve this problem, a law was enacted in 2008 [9] to replace the less binding agreement between The Ministry of Health and SALAR and to oblige the county councils to invite newly arrived asylum seekers to an HA. The intent of the law was to increase the proportion of asylum seekers being assessed, but it failed to do so. National annual statistics from when the law was launched in the year 2008 up until 2015 show that the average proportion of asylum seekers being assessed has remained around 50% [13, 23, 24].

### Policies and regulations

Because the Swedish counties vary significantly in regard to geographic size, location, population, economy, etc., it is not to be expected that healthcare services are organized and prioritized in exactly the same way throughout the country, which is also in congruence with the legal autonomy that the counties have. However, there are universal rights, values, and principles that must be considered and applied regardless of these differences [12, 25–27]. Similarly, compliance to national laws and regulations on, for example, healthcare professionals’ qualifications, quality of treatment and care, standards for documentation, performance monitoring, and a system for non-conformances is a must for all healthcare services [15]. These national regulations are interpreted and constitute the basis for regional and local policies that aim to ensure healthcare of high quality and safety. In this study, we found that 217 (68.8%) of the healthcare professionals reported having such steering documents at the county level to guide the HA in terms of content and performance.

Although many respondents seem to be familiar with existing policies, there was a considerable number that indicated a lack of awareness of such policies. There are reasons to believe that this, in a broad sense, might negatively influence the HA process from the invitation, through the assessment itself, and into the follow up.

### Organization and structure

An increasing administrative workload for doctors and nurses in the public sector has for some time been discussed and questioned [28, 29]. In this study, we found that some county councils had established a more formal centralized support function at the county level in order to ease the clinicians’ burden in terms of administration, accounting, legal, and policy matters. We found this to be a valuable resource that also might harmonize the healthcare centers’ routines in relation to HAs, improve the communication with the migration authority, and contribute to improved data quality for the national statistics on HAs among asylum seekers. However, this resource was known to only one third of the respondents working as clinicians at the healthcare centers. Our data do not reveal the reason for this, but insufficient communication from central offices to the periphery is likely to be the reason. We believe this is a waste of resources that would have benefited the nurses and GPs, and not least the asylum seekers in the end. Though 19 (95.0%) of the administrators indicated that they had a centralized administrative support unit in their county, only 192 (61.0%) clinicians also specified having similar administrative support. This does not give a depiction of a coherent system.

Among the examples presented on how different counties have organized their HA services and whether this is related to the number of asylum seekers being assessed at each site, one county appeared different from the others. That particular county had established a centralized healthcare center from where mobile teams reached out to all parts of the county where asylum seekers lived. Our preliminary conclusion is that we might have identified a unique and successful way of organizing the HA services targeting asylum seekers in one of Sweden’s counties.

### Competencies, responsibilities, and support

Nurse practitioners encompass a significant part of the healthcare professionals working in primary healthcare targeting refugees and asylum seekers [30, 31]. From this study, we have learned that nurse practitioners are also primarily responsible for carrying out HAs on asylum seekers in Sweden, though often in cooperation with GPs.

Working in a primary healthcare setting with asylum seekers from around the globe requires skills, experience, and sensitivity beyond what can be taught in basic training at nursing or medical school. The general frame of reference, not least in relation to issues concerning health, prevention, treatment, and the healthcare system, is for the most part vastly different between caregivers and the asylum seekers [11, 32, 33]. Postgraduate cross cultural health training, professional work experience abroad, or having personal experience with migration might increase the likelihood of an encounter characterized by respect, empathy, and trust [34, 35]. Although some respondents in this study did have some kind of postgraduate training related to migrants’ health, most did not, and 14% felt that they did not have the necessary competence in this area. However, almost one in five of the healthcare personnel had experience from professional work abroad within the healthcare sector. This might indicate that nurses and GPs with such experiences actively look for positions in a cross cultural healthcare setting in Sweden, assuming that their experience will be recognized and appreciated [34]. In a similar way, we believe that professional experience will benefit asylum seekers who undergo the HA. Approximately 50% of both the administrators and the healthcare professionals had many years of experience and had been working with asylum seekers for five years or longer, which indicates continuity and a limited staff turnover.

No matter how professionally skilled or experienced a nurse or a GP is, the need for a second opinion from a colleague, sharing experiences and good examples, and discussing the same will at some point be crucial. This is especially so when working on a small team or even alone in remote areas, working in a cross-cultural and bi-lingual setting, and dealing with complex and sensitive matters related to health. To some extent, more or less informal professional networks might serve as such a resource. However, relatively few respondents, especially among administrators, were part of such a network.

### The process and content of the health assessment

Cross cultural communication and information is in general considered to be challenging, especially when it comes to matters related to health [36, 37]. From this study, we learned that two thirds of the respondents, among the nurses and GPs, invited the asylum seekers to the HA by means of a written invitation in Swedish. It should be obvious that communicating in Swedish to newly arrived asylum seekers is not the best way to promote the HA as a positive offer, nor will it increase the number asylum seekers being assessed.

The aims of the HA are to identify health problems in asylum seekers and other migrants and to carry out effective measures of infectious disease prevention and control. It also aims to give the newcomers information about the Swedish healthcare system. The results from this research shows that these essential three areas of identification, prevention, and control are covered, but with substantial variations among the respondents as well as between the three focal areas. Other studies have revealed that asylum seekers frequently consider the content of the HA to be only an HIV test, which to some is linked to fear and bad feelings [11, 38, 39]. The result from this study confirms this picture because administering an HIV test was reported by our informants to be the most common component of the HA. However, important aspects related to the HIV test, such as information on how to prevent HIV, having information and booklets on HIV and other STIs available at the clinic, and checking on other sexual health issues within the HA were reported to be much less common. Asylum seekers have been described as commonly suffering from stress-related mental health problems [40], and if the HA does not consider the individual asylum seekers’ health needs, which seems to be the case according to our respondents, it is likely that mental health problems among this population will be left unattended. This is of course worrisome, especially when this study shows that access to psychiatrists and psychiatric nurse consultants, either by phone or on site, was rated the most insufficient in comparison to other specialists. In order to ensure that the content of the HA mirror the prevalence in burden of disease it is important that it is guided by evidence-based medicine.

### Performance monitoring

Performance monitoring is considered to be an important process in order to secure and develop quality in service and production, which also applies to the healthcare sector [41]. In this research, we focused on two issues related to performance monitoring and HAs. The first was crucial lead times, from when the asylum seeker arrives in Sweden until the HA is carried out, and the second was the estimated proportion of asylum seekers having undergone the HA. The estimated lead times show an almost even distribution, even when comparing the answers from the administrators to those of the healthcare personnel. The proportion of respondents who indicated “Do not know” and “missing” was 40–50% depending on the particular lead time. The result of the estimated proportion of asylum seekers who underwent the HA was even more discouraging. Only 40% of the administrators were able to approximately estimate the proportion of asylum seekers who had an HA carried out during the previous year in the county as a whole. The corresponding figures for the clinicians were as low as 19.3%. They were asked to estimate the proportion of asylum seekers who had an HA carried out during the previous year in the local healthcare center, and this result indicates a weak performance monitoring system or low compliance to an existing system.

### Strengths and limitations of the study

The method chosen for this research was an exploratory quantitative descriptive design applying a cross-sectional survey based on two structured questionnaires. We found the method used to be appropriate to capture the views from both administrators and healthcare professionals in relation to the aim of the study, namely to identify variations in policies and implementation of HAs for asylum seekers and how organizational differences might influence the number of asylum seekers being assessed. Because the contents of the two questionnaires were thematically similar, this made it possible to analyze to what extent there was a congruence between the two groups of respondents. Although not, per definition, using a mixed method, the fixed response alternatives were complemented with a few open-ended questions that gave us a deeper understanding of the organization and structure of the HAs.

The data collection for this study was conducted in 2010, which means that the results reflect the situation at that time. It is likely that policies and the organization of the healthcare related to HAs for asylum seekers have changed, at least to some extent, since then. However, from the national statistics we can conclude that the proportion of asylum seekers undergoing HAs has not increased during this period of time.

The relatively high number of non-responders led to a thoughtful analysis of missing questionnaires, which were identified and explained as an over coverage. For some questions, a relatively large number of internal missing answers was seen. Further analysis showed that such questions might not have been clear enough or that the response alternative “Do not know” should have been provided in order to prove what we do suspect, namely that the missing values might represent the respondents´ inability to respond to the question rather than unwillingness to respond.

## Conclusion

This paper has highlighted structural differences, strengths, and weaknesses among the Swedish counties in the health-related reception of newly arrived asylum seekers. The main finding is that there seems to be no coherent national system for carrying out HAs on asylum seekers in Sweden. The structures, organizations, processes, and outcomes vary between the counties, and there appear to be several reasons for the low coverage of HAs. Most importantly, we found that the number of healthcare centers in each county that carried out HAs on asylum seekers varied considerably regardless of the size of the county. Most commonly the HAs were carried out in ordinary health centers, although some counties presented alternative solutions on how to organize the HAs, among them one having mobile health teams in addition to a centralized healthcare center. In order to ease the burden of administration, etc., for health professionals carrying out HAs on asylum seekers, several county councils have established a centralized support function. Nevertheless, this important resource was not known to the majority of the healthcare personnel, which can be seen as a waste of resources. Last but not least, we argue that there is a need for improved performance monitoring on the part of the healthcare system that carries out HAs on asylum seekers.

This study might be seen as a baseline that needs to be followed up by other studies. Additionally, further research should focus on what seems to be a successful structure and organization in one of the participating counties, namely the use of mobile health teams.

## Abbreviations

EBO: Swedish: Eget BOende. English: Independent living
EU: European Union
GP: General practitioner
HA: Health assessments
HIV: Human immunodeficiency virus
SALAR: The Swedish Association of Local Authorities and Regions
STI: Sexually transmitted infections
TB: Tuberculosis
